# Can palmar creases serve as landmarks for the deeper neuro-vascular structures?

**DOI:** 10.1007/s00276-013-1211-4

**Published:** 2013-10-11

**Authors:** M. Kwiatkowska, T. Jakutowicz, B. Ciszek, J. Czubak

**Affiliations:** 1 Department of Descriptive and Clinical Anatomy, Centre of Biostructure, Research Medical University of Warsaw, Chalubinskiego 5, 02-004 Warsaw, Poland; 2 Department of Orthopaedics, Paediatric Orthopaedics and Traumatology, The Medical Centre of Postgraduate Education, Gruca Teaching Hospital, Konarskiego 13, Otwock, 05-400 Warsaw, Poland

**Keywords:** Palmar creases, Hand anatomy, Wrist anatomy, Palmar cutaneous branch of median nerve, Palmar cutaneous branch of ulnar nerve, Superficial palmar arch, Berrettini’s communicating branch

## Abstract

**Purpose:**

The aim of this study was the examination of the superficial anatomy of palmar creases and their relation to deeper neuro-vascular structures.

**Methods:**

Four creases: distal wrist flexion crease, thenar crease, proximal palmar crease and distal palmar crease were evaluated with reference to the following structures: palmar cutaneous branch of median nerve, palmar cutaneous branch of ulnar nerve, the nerve of Henle, transverse palmar branches from ulnar nerve, recurrent motor branch of median nerve, radial proper palmar digital nerve to the index and the ulnar proper palmar digital nerve to the thumb, Berrettini’s communicating branch, ulnar nerve and artery, superficial palmar arch. We performed dissections of 20 cadaveric upper limbs derived from a homogenous Caucasian group. In our study we measured the location of surgically important structures with reference to palmar skin creases.

**Results:**

Among the other observations we noticed that the palmar cutaneous branches of the median and ulnar nerves were located at least 0.5 cm away from the thenar crease. The superficial palmar arch was found between the thenar and proximal palmar crease and never crossed the proximal or distal palmar creases.

**Conclusions:**

These anatomical dissections will provide reference material for further ultrasound studies on the arrangements of neuro-vascular structures in reference to superficial palmar creases.

## Introduction

The palmar creases have been the subject of interest to scientists for centuries. Nevertheless, there are very few current references addressing the anatomy of palmar creases and its clinical significance. So far in the literature there have been descriptions of the palmar creases in reference to osseous structures or genetically inherited disorders [5, 7, 28]. Although the surgical approach to the palmar surface of the hand is usually straightforward, it may be occasionally a challenging task. There are several small cutaneous branches of nerves that are at risk when approach is being made to operate the hand. Several different incisions have been proposed to avoid the damage of these structures [1, 3, 6, 10, 16, 21, 24, 25, 29, 33]. In our study for the first time we decided to use the natural palmar lines-creases as a guide to localise precisely the important neuro-vascular structures.

The aim of this study was to examine the superficial anatomy of palmar creases and their relation to deeper structures. Four palmar creases were evaluated: distal wrist flexion crease, thenar crease, proximal palmar crease and distal palmar crease. The anatomy of palmar skin creases was evaluated with reference to the following structures: palmar cutaneous branch of median nerve (PCBMN), palmar cutaneous branch of ulnar nerve (PCBUN), the nerve of Henle, transverse palmar branches from ulnar nerve, recurrent motor branch of median nerve, the ulnar proper palmar digital nerves to the thumb, the radial proper palmar digital nerve to the index, Berrettini’s communicating branch, ulnar nerve and artery and superficial palmar arch.

## Materials and methods

**Fig. 1:**
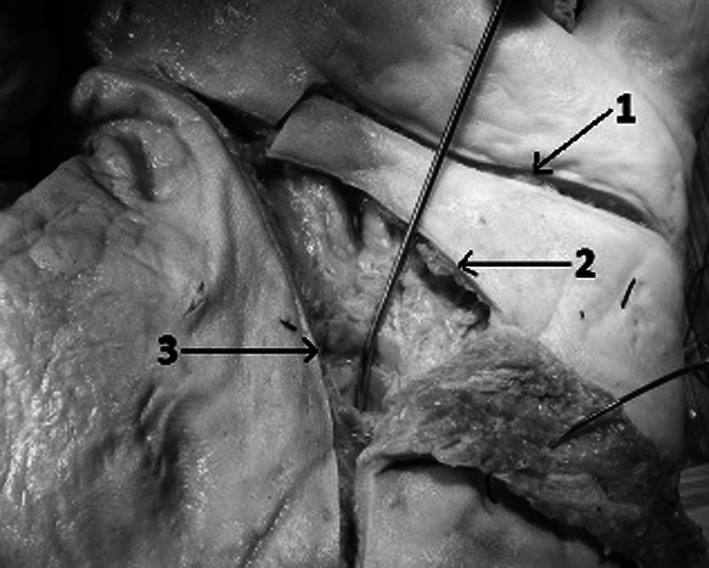
Palmar surface of the left hand. Incisions following the natural palmar creases: *1* distal palmar crease, *2* proximal palmar crease, *3* thenar crease. The probe is inserted deep to the flexor retinaculum

**Fig. 2:**
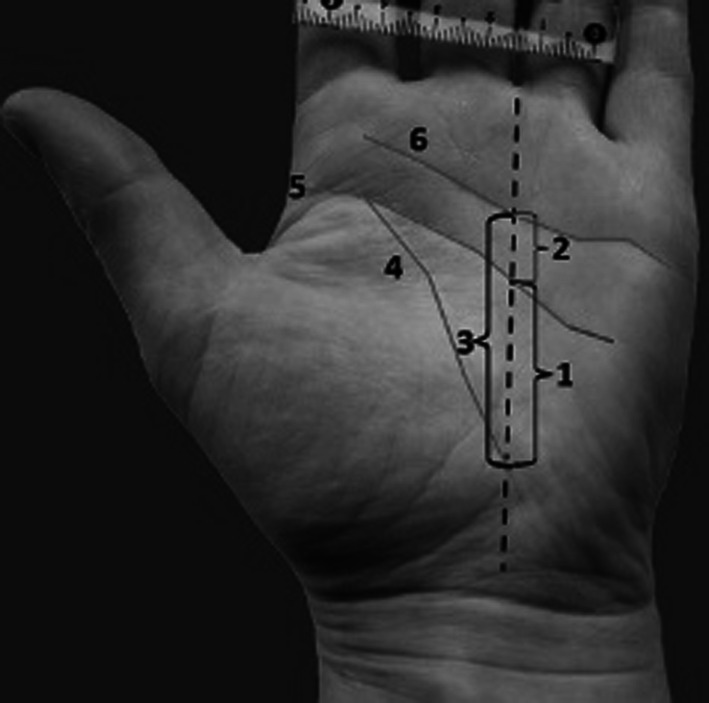
Distances between the creases. Measurements performed along the radial axis of the ring finger. *1* Distance between the thenar crease and the proximal palmar crease, *2* distance between proximal and distal palmar crease, *3* distance between the thenar and distal palmar crease, *4* thenar crease, *5* proximal palmar crease, *6* distal palmar crease

We performed dissections of 20 cadaveric upper limbs (11 right and 9 left). The specimens were obtained from the Department of Anatomy at the Medical University of Warsaw, Poland. Twelve limbs were fixed with formaldehyde solution and eight were fresh frozen. All specimens were derived from a homogenous Caucasian group. The dissections of the palmar surface of hands were performed with the aid of surgical microscope with magnification 2–5.5×. The incisions were performed within the natural palmar creases from the proximal to the distal end of the crease (Fig. 1). In the case of the distal wrist creases and the distal palmar creases the dissection was begun from the ulnar side of the crease. The subcutaneous fat was very thin under these creases and made the beginning of dissection much easier. In the first step of our work we were able to separate the palmar skin from the underlying subcutaneous fat leaving this layer intact. This was essential for cutaneous nerves identification and preservation during dissection. The palmar creases and the deeper structures, listed above, were identified and documented with a digital camera (Konica Minolta Dimage A200 8MP with Anti-shake 7× optical zoom, Korea) and analysed using a computer system (MultiScanBase v.13.08; Computer Scanning System, Warsaw, Poland). The measurements were taken from the photographs of the dissected specimens. Each picture contained a 5-cm-long ruler. The scale tools for the measurements were manually calibrated. The distances from the thenar crease to the proximal palmar crease and the distal palmar crease were measured along the radial axis of the ring finger (Fig. 2). The measurements of the structures crossing the distal wrist crease were performed from the ulnar to the radial side of the hand. The results of measurements were automatically transferred to the database of the MultiScanBase.

## Results

### Thenar crease

The thenar crease usually intersects the lateral side of the proximal palmar crease and curves obliquely across the palm to intersect the distal wrist crease near the wrist centre. The mean length of thenar crease was 7.37 cm (range 5–10.7 cm). The mean distance from thenar crease to proximal and to distal palmar crease evaluated along the radial axis of the ring finger (Fig. 2) was 0.97 cm (range 0.7–1.3 cm) and 1.58 cm (range 1.2–2.1 cm), respectively.

**Fig. 3:**
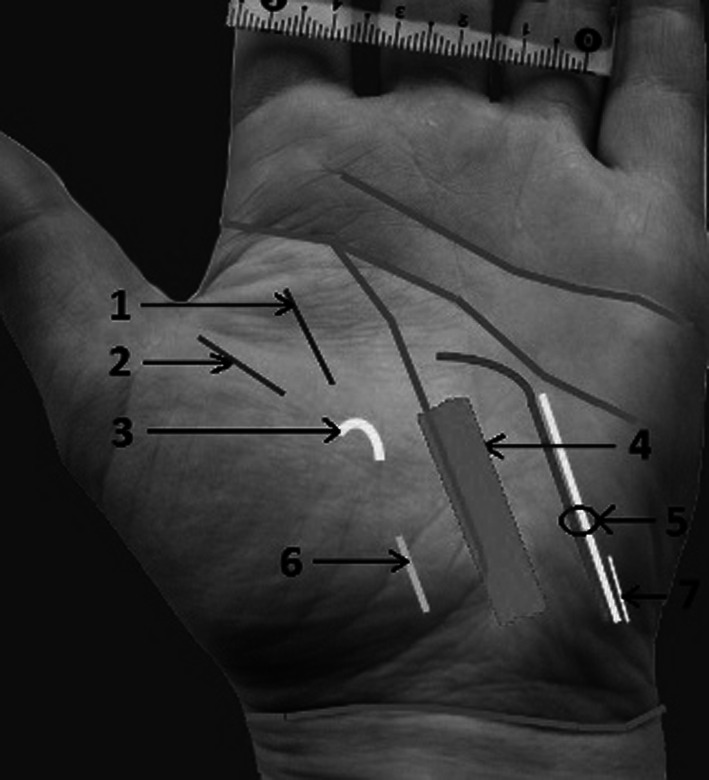
Distances between creases, nerves, and arteries: *1* radial proper palmar digital nerve to the index, *2* ulnar proper palmar digital nerve to the thumb, *3* recurrent motor branch of the median nerve, *4* carpal tunnel, *5* ulnar nerve, artery and nerve of Henle, *6* palmar cutaneous branch of the median nerve (PCBMN), *7* palmar cutaneous branch of the ulnar nerve (PCBUN)

The thenar crease, in our dissections, was never crossed by any cutaneous or motor branches of nerves. These structures were always located a few millimetres away from the thenar creases (at least 5 mm). The only structure to be found within the course of the thenar crease is the carpal tunnel (Table 1; Figs. 1, 3).

**Table 1:** Mean distances from thenar crease to important neuro-vascular structures of the wrist

Palmar cutaneous branch of the ulnar nerve (PCBUN)	Palmar cutaneous branch of the median nerve (PCBMN)	Recurrent branch of the median nerve	Branch of the median nerve to the index	Ulnar nerve, artery and the nerve of Henle	Carpal tunnel
2.12 cm (0.7–3)	1.1 cm (0.7–1.4)	1.09 cm (0.6–2.3)	1.87 cm (0.5–3.5)	1.71 cm (1–2.3)	0.43 cm (0–0.8)

### Superficial palmar arch

**Fig. 4:**
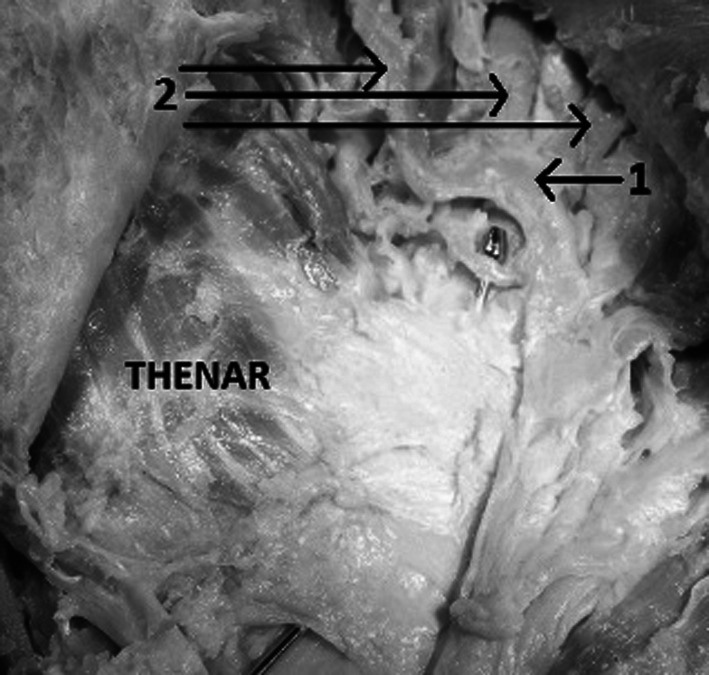
Dissected palm of the left hand: *1* superficial palmar arch, *2* common palmar digital arteries. The probe is inserted under the flexor retinaculum

**Fig. 5:**
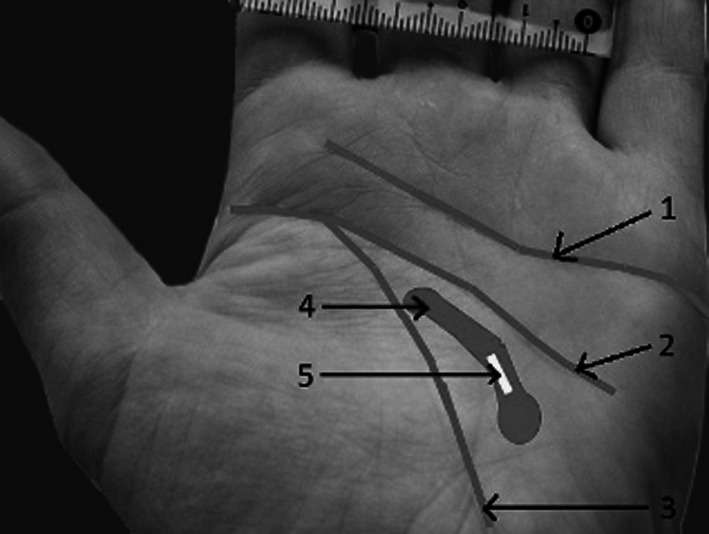
Distance from palmar creases to the superficial palmar arch and Berrettini’s communicating branch: *1* distal palmar crease, *2* proximal palmar crease, *3* thenar crease, *4* superficial palmar arch, *5* Berrettini’s communicating branch

The superficial palmar arch (Fig. 4) is located just beneath the palmar fascia and on top of the superficial tendons and may be complete or incomplete. It is located between thenar crease and proximal palmar crease. The distance from superficial palmar arch to the thenar crease was measured in three points of the arch: radially to the thenar crease (distal part of the arch), in the max convex of the arch, ulnarly to the crease (proximal part of the arch). The superficial palmar arch never crossed the level of proximal palmar crease. Moreover, the Berrettini’s communicating branch was located along the course of the superficial palmar arch and was usually located deep to the arch. In our study the superficial palmar arch was located between the thenar and the proximal palmar creases (Table 2; Fig. 5).

**Table 2:** Distance from the superficial palmar arch to the thenar crease measured in three points of the arch

Distal part of the arch	0.51 cm (0.02–1)
Convex part of the arch	0.72 cm (0.4–1.2)
Proximal part of the arch	1.07 cm (0.8–1.7)

### Berrettini’s communicating branch

**Fig. 6:**
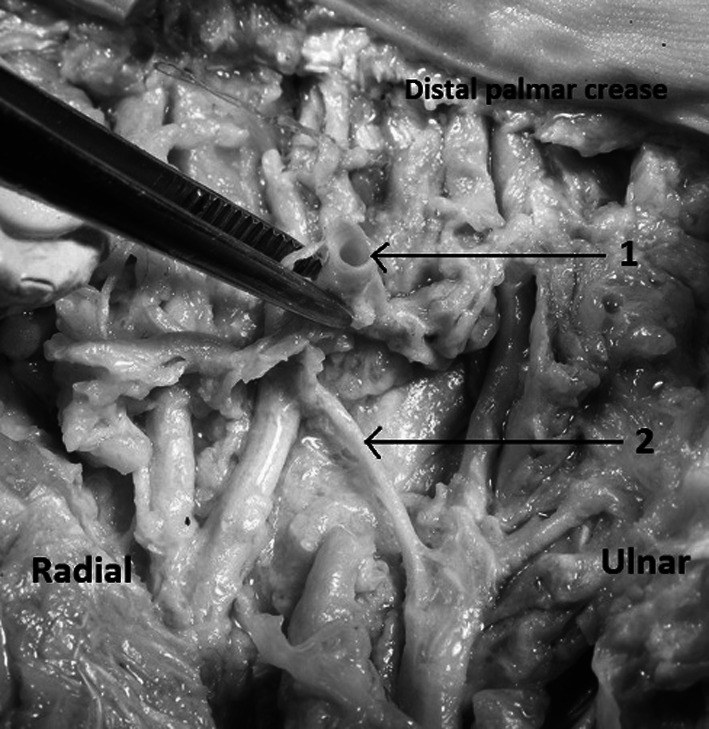
Dissected palm of the left hand: *1* superficial palmar arch, cut, *2* Berrettini’s communicating branch *3* distal palmar crease

**Fig. 7:**
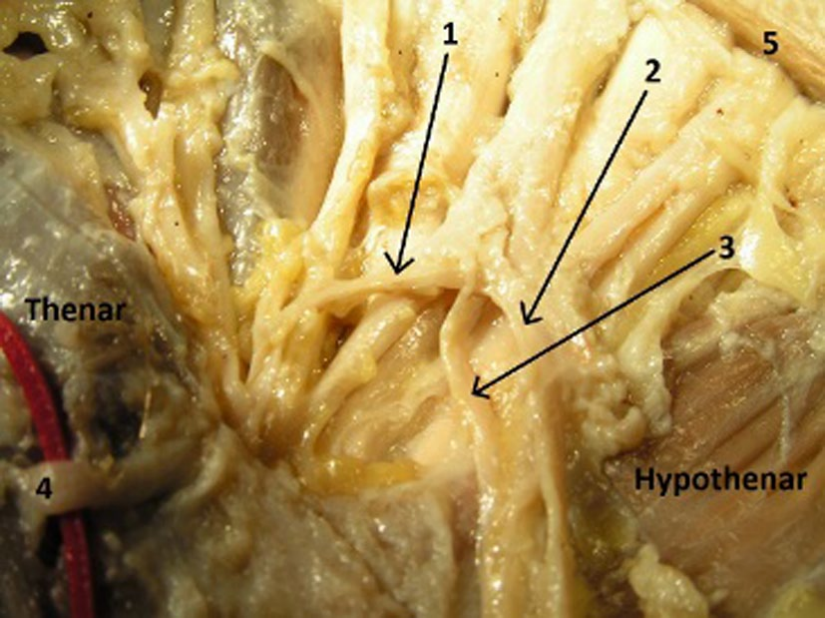
Dissected palm of the left hand: *1* Berrettini’s communicating branch, *2* ulnar nerve, *3* ulnar artery, *4* recurrent branch of median nerve, *5* distal palmar crease

The communicating branch between the median and ulnar nerve, known as the Berrettini’s communicating branch (Fig. 6), was also examined. This branch courses from the common digital nerve in the fourth interosseous space (ulnar nerve origin) to the common digital nerve in the third interosseous space (median nerve origin). Its course often parallels the superficial palmar arterial arch. In two cases this nerve was present in a plexiform nature. This communicating branch was perforated by the superficial palmar arch (Fig. 7). In the other specimens the Berrettini’s communicating branch of ulnar nerve was located deep to the superficial palmar arch.

The length of the branch was an average of 0.94 cm (range 0.8–1.2 cm).The distance from the thenar crease to the communicating branch was measured at two points:

1. At the ulnar end of the nerve.
2. At the median end of the nerve.

The superficial palmar arch and the Berrettini’s communicating branch were always found between the thenar crease and the proximal palmar crease (Table 3; Fig 5). These structures never crossed the line of the proximal palmar crease.

**Table 3:** Distance from the thenar crease to the Berrettini’s branch measured from both ends of the branch

From ulnar end	1.1 cm (0.7–1.3)
From median end	0.74 cm (0.4–1.3)

### Nerve of Henle

**Fig. 8:**
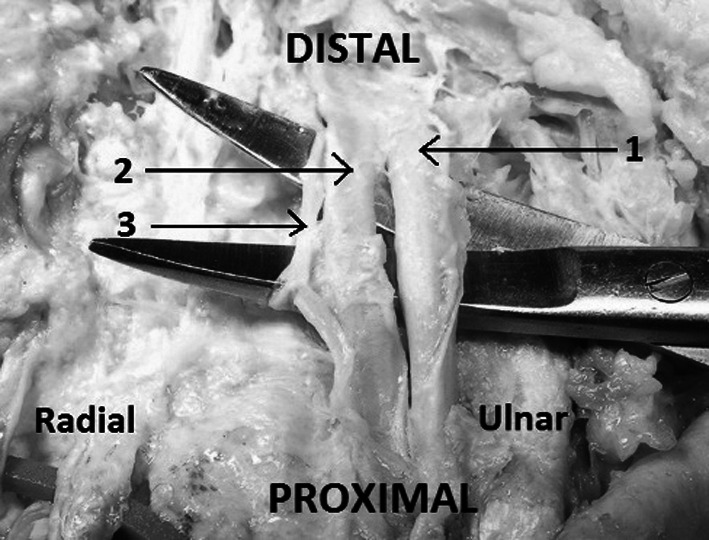
Dissected palm of the left hand: *1* ulnar nerve, *2* ulnar artery, *3* nerve of Henle

The triad of ulnar artery, nerve and nerve of Henle (Fig. 8) was also evaluated with reference to thenar crease.

The distance of this triad to thenar crease was an average of 1.71 cm (range 1–2.3 cm). The nerve of Henle, the sympathetic nervi vasorum of the ulnar artery, gives additional sensory innervations to the palm. The nerve lies alongside the artery and then branches off into smaller nerves alongside the superficial palmar arch.

### Distal wrist flexion crease

Although there are usually three wrist flexion creases, only the distal crease is of sufficient consistency to be used as a reliable landmark. The length of distal wrist crease was an average of 5.95 cm (range 4–7.5 cm).

**Fig. 9:**
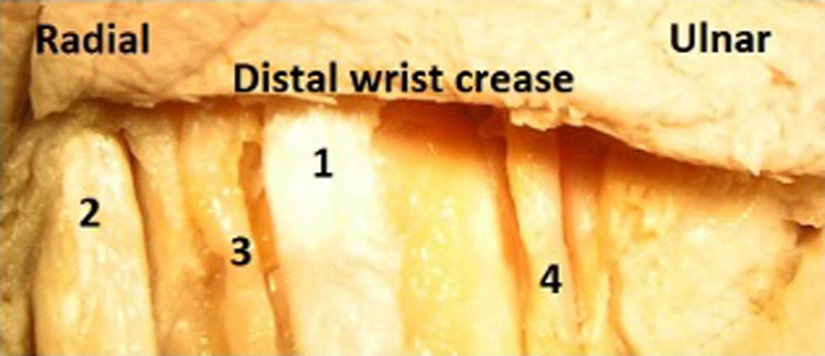
Palmar surface of the left hand. Dissection follows the distal wrist crease: *1* *PL* palmaris longus tendon, *2* *FCR* flexor carpi radialis tendon, *3* *PCBMN* palmar cutaneous branch of the median nerve, *4* *PCBUN* palmar cutaneous branch of the ulnar nerve

The location of anatomical structures crossing the distal wrist crease and their arraignment was also evaluated (Fig. 9).

Flexor carpi radialis tendon was located an average of 1.4 cm (range 0.7–2.1) from radial side of the hand crossing the line of the distal flexion wrist crease. Flexor carpi ulnaris tendon was identified an average of 1.35 cm (range 1.3–1.4 cm) from ulnar side of the hand. Palmaris longus tendon was absent in two cases and when present located an average of 2.95 cm (range 2.6–3.3 cm) from ulnar side of the hand and an average of 2.9 cm (range 2.4–3.3 cm) from radial side.

Ulnar artery and nerve with nerve of Henle was detected an average of 1.6 cm (range 1.2–1.8 cm) from ulnar side of the hand. The nerve of Henle was present in 100 % of specimens.

PCBMN was located an average of 3.11 cm (range 2.6–3.7 cm) away from radial side of the hand.

PCBUN was located an average of 3.82 cm (range 2–5.8 cm) away from radial side of the hand.

### The proximal and distal palmar creases

The length of proximal palmar crease was an average of 3.54 cm (range 0.7–7.7 cm) and the length of distal palmar crease was of an average of 4.42 cm (range 1.3–7.0 cm).

We also measured the distance from proximal to distal palmar crease along the radial axis of the ring finger and it was an average of 0.87 cm (range 0.6–1.0 cm) (Fig. 2).

We evaluated the distance from superficial palmar arch and Berrettini’s communicating branch to proximal and distal palmar creases.

The distance from superficial palmar arch to proximal palmar crease was an average of 1.33 cm (range 0.6–1.7 cm) and to the distal palmar crease an average of 2.17 cm (range 1.9–2.7 cm).

The distance from the Berrettini’s communicating branch to the proximal palmar crease was an average of 1.35 cm (range 0.6–2.1 cm) and to the distal palmar crease an average of 2.21 cm (range 1.6–2.7 cm).

## Discussion

Palmar creases, which are defined as the skin histologically attached to an underlying structure, are formed during the foetal period. The main creases of the palm may be morphologically constant, but with regard to length and position, they vary from person to person. The palmar creases naturally grow and develop with hand. They are unique for every human being. Palmar creases form at about 12 foetal weeks, although spontaneous hand movement does not occur before 11.5 foetal weeks. Therefore, although foetal hand movement may influence the development of the creases, genetic factors are considered most important to their formation [7, 19]. Our understanding of the creases and their values is yet incomplete. The gaps in our insight limit the possible interpretations and practical applications of the knowledge gained from the studies of various aspects of flexion creases.

Four palmar creases were evaluated in present study: distal wrist flexion crease, thenar crease, proximal palmar crease and distal palmar crease. These creases were consistently present in our specimens. We performed dissections of twenty cadaveric limbs from which twelve were fixed with formaldehyde solution and eight were fresh frozen. We are aware of the existence of some limitations to the present study. This anatomic study was performed in nonliving tissue and thus in vivo conditions could not be examined. Moreover, the cadaveric post mortem processing might have altered the neuro-vascular relationships. We do feel, however, that our sample of specimens preserved in two different conditions reflects the broad spectrum of patients encountered in a hand surgery practice.

In 1953, the most famous surgical landmark of hand known as the Kaplan’s [15, 17] cardinal line was described and used worldwide. Kaplan’s description of his landmark was a line “drawn from the apex of the interdigital fold between the thumb and index finger toward the ulnar side of the hand, parallel with the middle crease of the hand” The middle crease of the hand is the crease located between the distal palmar crease and the thenar crease and is also known in the literature as the proximal palmar crease [9].

As the later studies show, the line may be presented and used in four different ways by hand surgeons [32]. This may lead to the unintentional damage of important cutaneous branches as well as the vascular structures such as superficial palmar arch [2, 11, 14, 23].

The Kaplan’s cardinal line has been used as a surface marker in various anatomical studies. Anand and Trzeciak [1] anatomically correlated the relationship of Kaplan’s cardinal line to the superficial palmar arch. They found the superficial palmar arterial arch was located an average of 10.4 and 11.8 mm from the radial and ulnar borders of the ring finger with standard deviations of roughly 4 mm for each measurement.

In our study the superficial palmar arch was located between thenar crease and proximal palmar crease, which is similar to results of Anand and Trzeciak.

Other investigators, including Kaplan himself, have described numerous variations to the cardinal line [4, 16, 17, 27]. Current textbooks, even pinnacle works on surgical approaches, differ in their portrayal of Kaplan’s cardinal line [13]. This ambiguity becomes important when one considers the complex neurovascular patterns in the volar hand [11, 14]. More importantly, since Kaplan’s cardinal line has been described as representing the surface correlate of the motor branch of the median nerve [13, 27], deep branch of ulnar nerve [4, 16, 27], distal extent of the transverse carpal ligament [4] and the superficial palmar arch [4, 16, 18, 27], it becomes paramount to accurately define the landmark. We believe that palmar skin creases can be helpful here and serve as important additional superficial landmarks to guide the surgeon.

In our anatomical study we examined and measured not only the palmar creases but also important neuro-vascular structures such as nerve of Henle, transverse branches of ulnar nerve and Berrettini’s communicating branch. The nerve of Henle was present in 100 % of our specimens. According to the literature the nerve serves as the nervi vasorum of the ulnar artery, and gives innervations to the forearm and palm, in addition providing sympathetic innervations to the artery [9, 29]. This nerve was traced along the ulnar artery and the superficial palmar arch and its branches: common palmar digital arteries.

Accidental damage to this nerve can occur especially in the case of iatrogenic injury of the superficial palmar arch. This is not a benign occurrence and can result in complex regional pain syndrome [31].

Multiple cutaneous nerves to the palm are noted from the ulnar nerve. Many of these nerves exited perpendicularly from the longitudinal direction of the ulnar nerve, thus prompting a description of them as transverse palmar cutaneous branches of the ulnar nerve. These nerves pierced the palmar carpal ligament to innervate the skin and subcutaneous tissue of the hypothenar eminence and midpalm, usually distal to that area innervated by either the nerve of Henle or the palmar cutaneous branch of ulnar nerve [9, 29, 34].

The communicating branch of ulnar nerve, known as the Berrettini’s communicating branch, connecting the third common palmar digital branch of the median nerve with the fourth common palmar and proper palmar digital branches of the ulnar nerve was present in 100 % of our specimens. The earliest illustrations of the communicating branch between the digital branches of the median and ulnar nerves were the Berrettini drawings in 1741 [8]. Studies on the incidence of Berrettini anastomosis suggested that it should be considered a normal structure rather than an anatomical variation as its incidence was found to be over 80 % [8, 20, 22].

In two cases this nerve was present in a plexiform nature. This Berrettini’s communicating branch was perforated by the superficial palmar arch. The unusual relationship of Berrettini anastomosis with the superficial palmar arch is very rare, and knowledge about such a variation is important when performing carpal tunnel release, flexor tendon surgery, Dupuytren’s fasciectomy and when dealing with arterial repairs and vascular graft applications in the hand [30].

Endoscopic and open carpal tunnel releases, even if performed meticulously, still carry the potential for complications. A survey of American Society for Surgery of the Hand (ASSH) members published in 1999 noted a myriad of injuries, including damage to the superficial palmar arch (SPA) in both types of releases [26]. The preciseness of the incision utilised is paramount in preventing iatrogenic injury and ensuring a good outcome. The limited visual field in CTR, more appreciable in the endoscopic method, attests to the importance of topographical markers to delineate underlying structures [12, 26, 31]. Incisions can then be planned taking into consideration potential areas of neurovascular vulnerability. Thus examination of the superficial skin markers can make it easier to plan the operation.

As we mentioned above our cadaveric study has some limitation, but in the future we are planning to expand it on living subjects by performing the ultrasound examination of palmar hand surface. We will try to localise ultrasonographically the neurovascular structures with their correlation to palmar skin creases.

We hope this future study will add to that anatomical one and will help us to use the palmar skin creases as the reliable topographical landmarks for planning the safe surgical incisions.

## Conclusions

The palmar creases may serve as landmarks for the deeper neuro-vascular structures.

The superficial palmar arch was found between the thenar and proximal palmar crease and never crossed the proximal palmar crease.

The thenar crease, in our dissections, was never crossed by any cutaneous or motor branches of nerves.

These anatomical dissections will provide reference material for further ultrasound studies on the location of the neuro-vascular structures in reference to superficial palmar creases.
